# Poxvirus Host-Range Determinants: SAMD9/9L and Beyond

**DOI:** 10.1146/annurev-virology-092623-104658

**Published:** 2025-05-22

**Authors:** Yan Xiang

**Affiliations:** Department of Microbiology, Immunology and Molecular Genetics, University of Texas Health Science Center at San Antonio, San Antonio, Texas, USA

**Keywords:** poxvirus host range, innate immunity, interferon, SAMD9/9L, tRNase, tRNA

## Abstract

The recent global spread of mpox virus, facilitated by a newly established human-to-human transmission mode, has rekindled interest in poxviruses and the molecular factors defining their host range. Poxviruses employ host-range factors, a subset of their immune evasion proteins, to overcome cell-intrinsic defenses in specific cell types or host species. Over the past decade, investigations of these factors have revealed previously unrecognized antiviral mechanisms and expanded our understanding of innate immunity. Among the key developments are the discovery of novel restriction factors, including SAMD9 and SAMD9L (SAMD9/9L), and expanded roles for established antiviral proteins such as IFITs, FAM111A, and ZAP. These advances not only clarify how poxvirus host range is determined but also offer valuable insights into the complexity and evolution of mammalian innate immunity. Here, I highlight new findings on poxvirus host-range determinants, with a particular focus on SAMD9/9L and the three distinct classes of poxvirus host-range factors that antagonize them.

## 1. INTRODUCTION

Poxviruses are large, double-stranded DNA (dsDNA) viruses that replicate entirely in the cytoplasm of infected cells and employ sophisticated strategies to modulate host responses (1). Within the poxvirus family, members of the *Orthopoxvirus* (OPXV) genus have had a profound effect on human health. Variola virus (VARV), the causative agent of smallpox, is one of the most lethal pathogens in history, and its eradication represents a major milestone in public health. More recently, mpox virus (MPXV, formerly known as monkeypox virus) has demonstrated human-to-human transmission and global spread beyond its endemic regions, renewing interest in this previously overlooked viral family (2). Several novel OPXV species have also been identified in human cases in recent years (3, 4), although their animal sources remain unknown, highlighting the complex and poorly understood ecology of poxviruses.

Beyond OPXVs, other poxvirus genera cause significant diseases in animals (5). For example, lumpy skin disease virus has threatened livestock industries worldwide. Myxoma virus (MYXV), lethal to European rabbits, was introduced into Australia and Europe to control rabbit populations. Over time, it became established in European rabbit populations, and a recent recombinant strain has caused outbreaks in a new animal host (6), exemplifying the evolutionary capacity of poxviruses to adapt to novel hosts.

Poxviruses collectively infect a broad taxonomic range of hosts, but individual poxvirus species often exhibit relatively narrow and distinct host ranges, sometimes differing markedly even among closely related viruses. For example, VARV is limited to humans, while MPXV infects a broader range of mammals, facilitating its persistence in wildlife reservoirs and spillover into new hosts. The molecular mechanisms underlying these differences are not yet fully understood, but host-range variations have long been exploited for vaccine development and oncolytic virotherapy. A prime example is modified vaccinia Ankara (MVA), which is generated by more than 500 serial passages of the parental vaccinia virus (VACV) in avian cells (7). MVA, which has lost 30 kilobases (kb) of its genome, can no longer replicate productively in mammalian cells and is used as a safe vaccine for smallpox and mpox. Similarly, avian poxviruses and some mammalian poxviruses with narrow host ranges have been harnessed as vaccine vectors or oncolytic agents (8).

**Figure 1:**
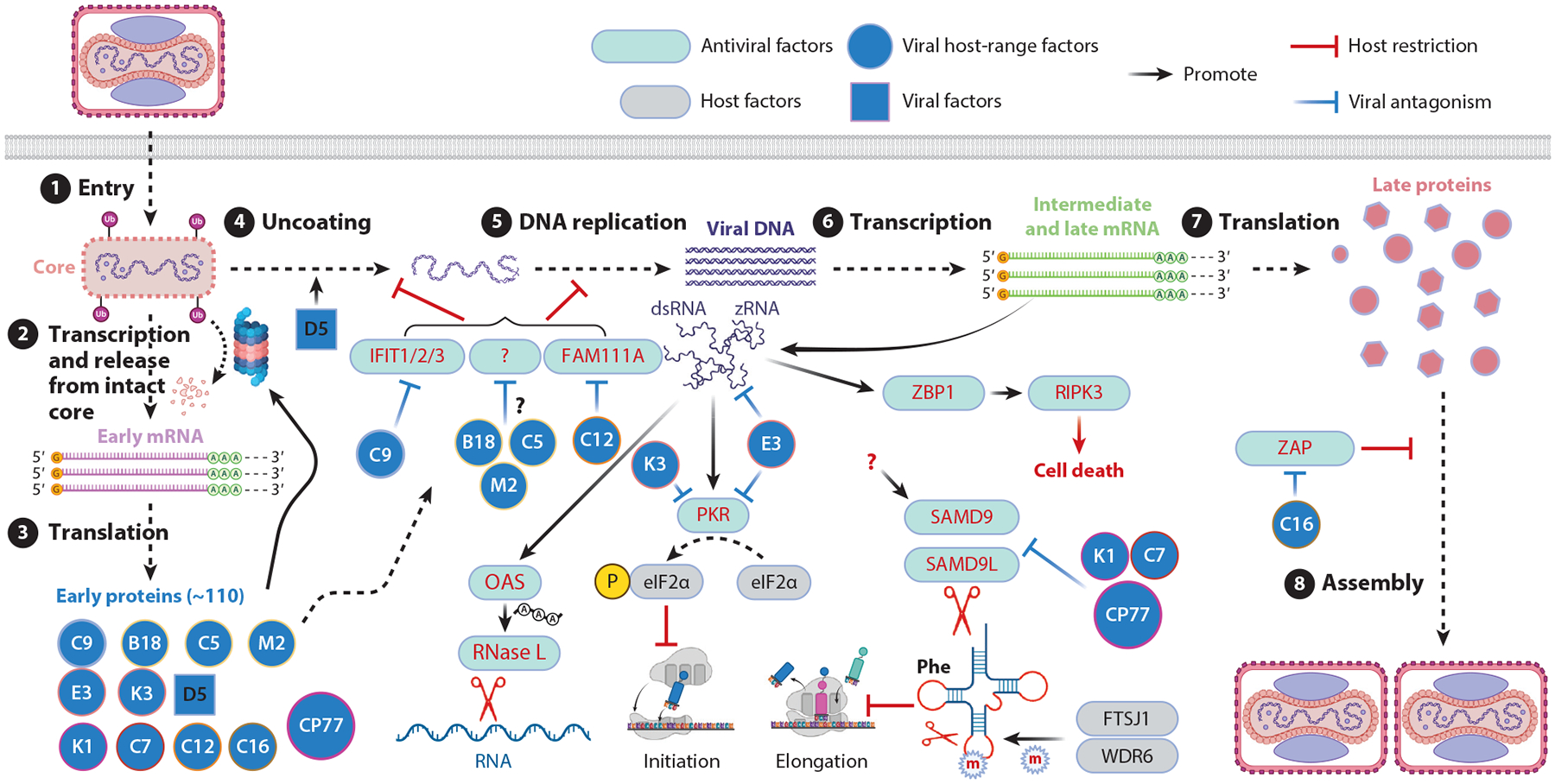
The interplay between host restriction factors and VACV host-range factors during the viral replication cycle in the cytoplasm. Major steps of the VACV life cycle are depicted and numbered: ① viral entry ② early mRNA synthesis ③ early protein synthesis ④, genome uncoating, ⑤ genome replication, ⑥ intermediate and late mRNA synthesis, ⑦ intermediate and late protein synthesis, and ⑧ virion assembly and morphogenesis. Host restriction factors targeting specific steps of the replication cycle are depicted as rectangles, while viral host-range factors that counteract these defenses are shown as circles. See Section 2 for a detailed description. Abbreviations: dsRNA, double-stranded RNA; m, methylation; mRNA, messenger RNA; OAS, oligoadenylate synthetase; PKR, protein kinase R; SAMD9/SAMD9L, sterile alpha motif domain-containing 9/L; VACV, vaccinia virus; ZAP, zinc-finger antiviral protein; ZBP1, Z-DNA-binding protein 1; zRNA, left-handed (Z-form) dsRNA. Figure adapted from images created in BioRender; Xiang Y. 2024. https://BioRender.com/l18z217.

Unlike many other viruses, where entry receptor specificity often determines host range, poxviruses can enter nearly all animal cells (9). Their ability to infect a host species depends on complex virus-host interactions at the cellular, tissue, and organismal levels (10). At the cellular level, the host range of poxviruses is primarily determined by their unique repertoire of viral host-range genes (11). These host-range factors are a subset of the broader repertoire of poxvirus immune evasion molecules, which include inhibitors of cytosolic DNA sensing, nuclear factor kappa B (NF-κB) activation, interferon induction, interferon effectors, and cell death pathways. Poxvirus immune evasion strategies have been comprehensively reviewed recently (12), so this review focuses on new insights into molecular determinants of poxvirus host range uncovered since the last major reviews on this topic appeared more than a decade ago (5, 11, 13). In particular, mammalian SAMD9 (sterile alpha motif domain-containing 9) and its paralog, SAMD9L, have emerged as critical restriction factors against poxviruses and are antagonized by three distinct classes of poxvirus host-range factors (14–17) (Figure 1). These proteins are a major focus of this review.

## 2. MOLECULAR DETERMINANTS OF POXVIRUS HOST RANGE

### 2.1. Overview of Poxviruses and Their Host Ranges

**Figure 2:**
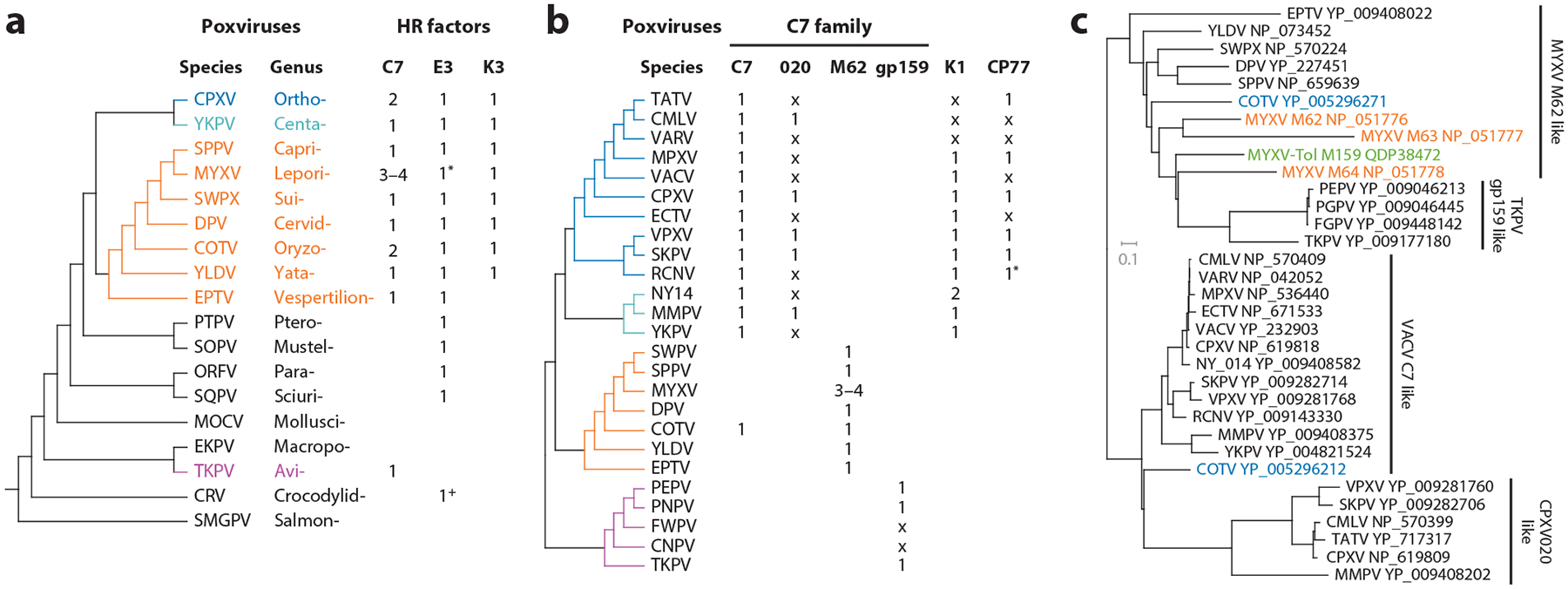
Poxvirus phylogeny and distribution of HR genes. (*a*) Phylogenetic tree of 18 *Chordopoxvirus* genera and the distribution of HR factors related to VACV C7, E3, and K3. Numbers represent the count of homologs identified in each virus species. Asterisks and plus signs denote partial homologs. The poxvirus suffix is omitted from genus names for simplicity. The *Chordopoxvirus* phylogeny is adapted from Ref. 120. (*b*) Phylogenetic tree of selected *Chordopoxvirus* genera and the distribution of HR factors related to SAMD9/9L inhibition (C7 family, VACV K1, and CPXV CP77). The C7 family is further divided into four clades represented by VACV C7, CPXV020 (20), MYXV M62, and TKPV gp159. Tree line colors correspond to the genus color in panel *a*. The x's and asterisk denote gene loss and partial homolog, respectively. (*c*) Phylogenetic relationships of poxvirus C7 family members, derived from multiple sequence alignment and constructed using the NCBI BLAST Fast Minimum Evolution Tree Builder. Four major clades are identified, represented by VACV C7, CPXV020, MYXV M62, and TKPV gp159. Virus names and protein accession numbers are displayed. Note that two C7 homologs from COTV (*blue*) are associated with different C7 clades. Three C7 homologs from MYXV are shown in orange, while the additional C7 homolog from MYXV-Tol is shown in green. Abbreviations: COTV, Cotia virus; CPXV, cowpox virus; HR, host range; MYXV, myxoma virus; NCBI BLAST, National Center for Biotechnology Information Basic Local Alignment Search Tool; SAMD9/9L, sterile alpha motif domain-containing 9/L; TKPV, turkeypox virus; VACV, vaccinia virus. Refer to Table 1 for additional abbreviations of virus names.

The *Poxviridae* family is divided into two subfamilies: *Entomopoxvirinae*, which infect insects, and *Chordopoxvirinae* (ChPV), which infect vertebrates. About a decade ago, 10 ChPV genera were recognized: OPXV, *Suipoxvirus*, *Capripoxvirus*, *Leporipoxvirus*, *Cervidpoxvirus*, *Yatapoxvirus*, *Parapoxvirus*, *Molluscipoxvirus*, *Avipoxvirus*, and *Crocodylidpoxvirus*. Since then, advances in virus discovery and next-generation sequencing have substantially expanded ChPV diversity to 18 genera (18) (Table 1; Figure 2a). Salmon gill poxvirus, the first fully sequenced fish poxvirus, is now classified under the new genus *Salmonpoxvirus*, a basal lineage within ChPV (19). Cotia virus (COTV) (20), isolated from sentinel mice in Brazil, has been designated as the genus *Oryzopoxvirus*. Several OPXV-related viruses, likely originating from rodents, are grouped in the new genus *Centapoxvirus* (CETV). Additionally, novel poxviruses isolated from various vertebrates have been placed into additional genera: *Mustelpoxvirus* (isolated from sea otters), *Pteropopoxvirus* (from little red flying foxes), *Vespertilionpoxvirus* (from big brown bats), *Sciuripox* (from red squirrels), and *Macropopoxvirus* (from kangaroos). The *Avipoxvirus* genus has grown in diversity, with related species identified in reptiles (21). Within the OPXV genus, novel species have been isolated from human or animal sources (3, 4, 22).

The natural host ranges of most poxviruses remain unknown; however, they are generally narrow and restricted to a single taxonomic genus. Nonetheless, some OPXV species exhibit broad host ranges. The OPXV genus includes VARV, MPXV, VACV, cowpox virus (CPXV), and camelpox virus (CMLV) (Figure 2b). Host ranges among OPXVs vary significantly: VARV and CMLV are highly host specific, limited to humans and camels, respectively, whereas VACV, MPXV, and CPXV have broader host ranges, increasing their potential for spillover into human populations. Rodents are frequently implicated as reservoirs for OPXVs. VARV, an exclusive human pathogen, is believed to have evolved from a rodent-borne ancestor (23). CPXV is carried by bank voles and striped field mice in western Europe (24, 25). In Brazil, VACV is thought to spread through wild rodents acting as reservoirs or intermediate hosts (26). MPXV is believed to be maintained in African rope squirrels (27).

### 2.2. Poxvirus Host-Range Genes

Poxvirus genomes consist of linear dsDNA ranging from ~128 to ~375 kb. The central region of the genome is relatively conserved, while the two terminal regions are more variable and confer unique characteristics to different poxvirus species. The central region harbors ~90 genes con served across all ChPVs, encoding proteins essential for viral entry, gene transcription, genome replication, and virus assembly (28).

The variable terminal regions contain genes that primarily mediate virus-host interactions. These genes encode secreted factors that bind complement proteins, cytokines, or interferons to prevent immune responses, as well as intracellular factors that inhibit immune signaling pathways, antiviral factors, or cell death (12). Some of these genes affect viral replication only in a subset of cells and are referred to as host-range genes. They have been identified primarily through studies of viral mutants defective in replication in specific cell types (11, 13, 29). Known poxvirus host-range genes have been classified into ~12 classes (13). Their distribution in the poxvirus family varies due to gene gain, loss, and recombination events. Canonical VACV host-range genes include *E3L*, *K3L*, *K1L*, *C7L*, and serpins (serine protease inhibitors) *SPI-1*, *SPI-2*, and *SPI-3*. [VACV genes are conventionally named according to genome fragments generated by HindIII digestion, labeled A through O, followed by an open reading frame number and orientation “L” or “R” (e.g., *C7L*). The protein product is typically referred to without L/R, e.g., C7. A unified OPXV gene numbering system was recently proposed (30).]

The functions of these host-range factors are not fully understood; however, many are known to counteract cell-intrinsic antiviral activities. Host-range factors frequently contain protein-protein interaction motifs, including ankyrin (ANK) repeats and the PRANC (pox protein repeats of ankyrin, C-terminal) domain (31). ANK repeats are rare in viruses outside the poxvirus family. Each ANK repeat consists of a helix-loop-helix-β-hairpin/loop module. Different ANK proteins assemble varying numbers of these repeats and possess diverse surface residues, enabling interactions with distinct host targets (32). The PRANC domain, a variant of the F-box motif, interacts with the E3 ubiquitin ligase complex for protein degradation (33).

The repertoire of poxvirus host-range genes—and their species-specific interactions with host targets—appears to strongly influence viral host range (34). In general, poxviruses encoding a larger number of these genes exhibit broader host tropisms. For example, CPXV retains the greatest number of host-range genes among OPXVs, correlating with its ability to infect a diverse array of host species.

### 2.3. Vaccinia Virus Host-Range Factors and Their Host Targets

Because poxviruses replicate robustly in the cytoplasm, they generate conspicuous pathogen-associated molecular patterns (PAMPs) and place heavy demands on cellular resources, creating multiple opportunities for host restriction. Consequently, poxviruses devote large segments of their genomes to counter host restrictions. Host restriction often becomes evident when viral mutants lacking specific countermeasures exhibit restricted host ranges. The discovery of host restriction factors against these viral mutants has been accelerated by the advent of high-throughput genetic tools, including genome-wide RNA interference screens (16, 35, 36). These studies have provided new insights into poxvirus cell tropism and host innate immunity. Below, I discuss key VACV host-range factors and their targets in the context of a single-cell viral life cycle (1, 37) (Figure 1).

#### 2.3.1. Viral entry and early gene expression.

VACV can enter cells either by direct fusion at the plasma membrane or via endocytosis and subsequent fusion with the endosomal membrane. Upon entry, the viral core, a stable structure that does not dissolve spontaneously, is released into the cytoplasm. Early messenger RNA (mRNA) synthesis begins immediately within the intact core, which contains a complete transcription apparatus for early gene expression, including capping and polyadenylation enzymes.

Roughly half of VACV's ~200 genes are expressed as early genes (38). The capped and polyadenylated early mRNAs are extruded through pores in the virion core into the cytoplasm for translation. Early viral proteins include inhibitors of intracellular sensors as well as factors required for uncoating, such as D5 (39). Because no specific receptors appear to be required for VACV entry and the genome remains shielded within the core during early gene expression, relatively few host restrictions act at this early stage.

#### 2.3.2. Genome uncoating and replication.

Core dissolution and genome uncoating, poorly understood processes that require both viral early proteins and host proteasomes, release viral DNA into the cytoplasm. The viral genome then serves as the template for DNA replication and post-replicative (intermediate and late) gene expression. Several host factors can inhibit genome uncoating and/or replication, including BAF (barrier-to-autointegration factor), IFIT1/2/3 (inter-feron induced protein with tetratricopeptide repeat 1/2/3), and FAM111A (family with sequence similarity 111 member A). BAF binds DNA and inhibits VACV genome replication unless inactivated by the poxvirus B1 kinase (40). (Because B1 is not a host-range factor, it is not covered here.)

Interferon-induced IFIT1/2/3 bind 5′-triphosphate or ribose-unmethylated capped ends of mRNA to inhibit translation (41). VACV C9 uses its N-terminal ANK repeats and C-terminal PRANC domain to bind IFITs and E3 ubiquitin ligase complex, respectively, targeting IFITs for proteasomal degradation (42, 43). A C9-deletion VACV mutant expresses early proteins but is blocked at the genome uncoating and replication step, suggesting IFITs may have additional antiviral mechanisms beyond translation inhibition.

FAM111A is a mammalian trypsin-like peptidase normally present in the nucleus, where it regulates DNA replication (44). Recent studies show that FAM111A can inhibit VACV replication by relocating to the cytoplasm and specifically targeting the viral DNA-binding protein I3 for degradation (45). VACV SPI-1, encoded by *C12L*, enters the nucleus of infected cells and inhibits FAM111A's peptidase activity. This inhibition prevents FAM111A from degrading the nuclear pore complex, thereby confining FAM111A to the nucleus.

Additional VACV host-range factors that act at this stage include B18, a protein with ANK repeats, and PRANC, which is a host-range factor for MVA (46). Interestingly, B18's function can be substituted by two other, unrelated VACV proteins, C5 and M2, both of which are absent in MVA (47). These mutually redundant proteins are required for VACV genome uncoating and replication in certain human cell lines but not in chicken cells, suggesting that they may target as-yet-unidentified host restriction factors that impede viral genome uncoating and replication.

#### 2.3.3. Intermediate and late transcription.

Genome replication triggers post-replicative transcription. Unlike early mRNAs, which possess defined 3′ ends due to recognition of specific transcription termination signals by VACV termination factor, post-replicative mRNAs are long and have heterogeneous 3′ ends. Overlapping transcripts form double-stranded RNA (dsRNA), which activates several well-characterized antiviral pathways, including the protein kinase R (PKR) and oligoadenylate synthetase (OAS)-RNase L pathways. PKR phosphorylates eIF2a, inhibiting the initiation of protein synthesis. OASs synthesize 2–5 oligoadenylates (2–5A), which bind and activate RNase L, resulting in the cleavage of ribosomal RNA and mRNA. Recent studies showed that VACV replication also generates Z-RNA, a left-handed (Z-form) dsRNA, which binds Z-DNA-binding protein 1 (ZBP1), leading to RIPK3-mediated cell death (48, 49).

VACV E3 contains an N-terminal Z-nucleic acid–binding domain and a C-terminal dsRNA-binding domain, which sequester Z-RNA and dsRNA, respectively (50–52). Additionally, VACV K3 is an eIF2a mimetic and functions as a pseudosubstrate inhibitor of PKR (53, 54).

#### 2.3.4. Translation of viral and host messenger RNAs.

Viruses rely entirely on the host cell's translational machinery for protein synthesis, making translational control a critical viral restriction mechanism. In addition to the well-characterized PKR pathway, mammalian proteins SAMD9 and SAMD9L (collectively referred to as SAMD9/9L) have been identified as critical restriction factors that inhibit protein synthesis (discussed in detail in Section 3). These factors are specifically targeted by three distinct classes of host-range factors, represented by VACV K1, VACV C7, and CPXV CP77 (discussed in detail in Section 4).

#### 2.3.5. Assembly and morphogenesis.

Virus assembly initiates in cytoplasmic factories following genome replication and post-replicative gene expression, which produce all virion components and regulatory proteins essential for virion morphogenesis. The assembly and morphogenesis of virions are complex processes that generate intracellular mature virions (MVs). Subsequently, some MVs are wrapped with Golgi-derived membranes to form intracellular enveloped virions, some of which are exocytosed as extracellular enveloped virions.

Replication of MVA in mammalian cells is restricted during the morphogenesis stage (7). This restriction can be partially alleviated by knocking down the zinc-finger antiviral protein (ZAP) (35). ZAP is known for inhibiting specific RNA viruses by binding to CpG-enriched motifs in viral RNA, thereby promoting RNA degradation or inhibiting translation (55). However, in the context of MVA replication, ZAP does not significantly affect viral DNA replication, mRNA expression, or protein synthesis. Instead, ZAP interferes with the later stages of MVA virion morphogenesis, resulting in the accumulation of defective viral particles with irregular dense cores (35).

VACV C16, a viral protein with a Bcl2-like fold, has been identified as an antagonist of ZAP. C16 directly binds to ZAP and sequesters it within cytoplasmic punctate structures (35). Remarkably, repairing both the SPI-1 (*C12L*) and *C16L* genes in MVA boosts viral replication to levels comparable to those observed in permissive chicken embryo fibroblasts (56), thereby fully defining the genetic basis of MVA's restricted host range.

## 3. SAMD9 AND SAMD9L

### 3.1. Discovery of Mammalian SAMD9 and SAMD9L as Restriction Factors Against Poxviruses

VACV *K1L* and *C7L* and CPXV CP77 were discovered in the 1980s as the first poxvirus host-range genes (57–59). VACV mutants lacking both *K1L* and *C7L* (VACV/K1^−^C7^−^) are unable to replicate in most mammalian cells due to a block in translating viral post-replicative mRNAs (57, 59–62). Despite their structural differences, K1, C7, and CP77 function equivalently to support VACV replication in many cell types. However, the restriction factors they target remained elusive for decades.

Early studies showed that K1 and CP77 bind various host proteins, such as ACAP2 (63, 64) or HMG20A (65), and inhibit NF-κB activation (66, 67). Yet, these interactions did not explain the host-range effect, and NF-κB activation proved irrelevant to host restriction (67).

Using VACV/K1^−^C7^−^ as the starting point, Meng et al. (61) surveyed C7 homologs from a wide variety of mammalian poxviruses, finding that many, including MYXV M62, fully substitute for C7. This indicates that the unidentified targets of C7 must be highly conserved in mammals. Furthermore, these host targets were determined to be interferon-stimulated genes (ISGs), as interferon treatment induces restriction of VACV/K1^−^C7^−^ in otherwise permissive human Huh7 or M CF-7 cells (68). A screen of a library containing more than 350 ISGs identified IRF1 (inter-feron regulatory factor 1) as an inhibitor of VACV/K1^−^C7^−^, suggesting that IRF1 can also induce the expression of the putative host restriction factors (69). Importantly, Liu et al. (17) found that MYXV M62 binds human SAMD9 (hSAMD9), which restricts an M62-deletion MYXV mutant. Although SAMD9 fits the profile of the restriction factor for VACV/K1^−^C7^−^, being an ISG that can also be induced by IRF1 (70), it was initially thought to be a specific target for M62 but not for C7 or K1, due to early difficulty in demonstrating SAMD9 binding to the VACV proteins (17). Eventually, Sivan et al. (16, 71) conducted a human genome-wide small interfering RNA screen for restriction factors against VACV/K1^−^C7^−^ and identified SAMD9 as the primary restriction factor, with FTSJ1 and WDR6 as minor contributors.

SAMD9 knockout (KO) in human cells completely removes the restriction of VACV/K1^−^C7^−^, but interferon induction or IRF1 overexpression in SAMD9 KO cells restores the restriction (15, 16). This led Meng et al. (15) to identify SAMD9L as the second restriction factor for VACV/K1^−^C7^−^. In many human cells, SAMD9 acts as the constitutive restriction factor, whereas SAMD9L requires interferon induction to reach sufficient levels for restricting poxviruses (15). In mice, which naturally lack SAMD9, SAMD9L serves as the constitutive restriction factor against poxviruses (15). VACV/K1^−^C7^−^ mutants are highly attenuated in SAMD9L-proficient mice but regain virulence in SAMD9L KO mice (15), demonstrating the critical role of SAMD9/9L as a host barrier for poxvirus replication and pathogenesis.

### 3.2. SAMD9 and SAMD9L Distribution in Host Species

SAMD9 and SAMD9L were initially noted in mammals, where a gene duplication event early in mammalian evolution gave rise to these paralogous genes (72). While some mammals have lost one paralog, all retain at least one, suggesting their essential yet partially overlapping functions. SAMD9/9L exhibit signs of positive selection, particularly in rodents, suggesting evolutionary pressures exerted by pathogen interactions (72). In humans, SAMD9 and SAMD9L are tandemly located on chromosome 7, sharing ~60% amino acid (aa) identity. They are cytosolic proteins expressed in many tissues (73), further inducible by interferons or IRFs (15).

Further sequence analysis has shown that SAMD9/9L homologs are widespread in both vertebrate and invertebrate animals but are notably absent in plants, arthropods, and nematodes (74). Independent duplications of the SAMD9/9L gene have occurred in vertebrates, particularly in bony fishes. Interestingly, bacterial homologs of SAMD9/9L have also been identified, though they share only the central core domains with animal SAMD9/9L proteins, and their functions are harder to predict.

### 3.3. Cellular Processes Affected by SAMD9 and SAMD9L

Before SAMD9/9L were identified as the restriction factors, it was already established that the restriction of VACV/K1^−^C7^−^ occurs at the stage of viral and host protein synthesis (57, 59–62), prompting studies to investigate cellular processes that might influence translation. Some noted enhanced activation of PKR and phosphorylation of eIF2a during infection with VACV mutants lacking *K1L* and *C7L* (61, 75). However, knockdown of PKR fails to restore protein synthesis or viral replication (68, 75), indicating that PKR activation and eIF2a phosphorylation are secondary to abortive infection rather than its primary cause. Even dual KO of PKR and RNase L fails to alleviate the restriction (71).

Other studies found that, during poxvirus infection, SAMD9 relocalizes to cytoplasmic granules containing dsRNA and stress granule markers such as G3BP, TIAR, and TIA1 and the translation factor eIF4G (71, 76, 77). However, KO of G3BP1/2 (71), TIAR, or TIA1 (76), key stress granule components, does not affect granule formation or VACV restriction, suggesting that these granules reflect downstream consequences rather than the root restriction mechanism. The failure of PKR, RNase L, or G3BP KO to rescue protein synthesis, coupled with the observed inhibition of both cap-dependent and internal ribosome entry site–mediated translation, suggested an atypical mechanism of translational inhibition by SAMD9 (71).

More recently, the abortive replication of M62-deletion MYXV was linked to increased expression of ISGs mediated by the cGAS-STING pathway (78). SAMD9 knockdown reduces this proinflammatory response, suggesting that SAMD9 may regulate cGAS sensing of DNA. However, it remains unclear whether SAMD9 is directly involved in cGAS signaling.

A significant challenge in studying SAMD9's function during abortive poxvirus replication is distinguishing primary causes from secondary consequences of reduced cellular or viral protein synthesis. For instance, enhanced PKR activation during infection may partially result from decreased levels of the VACV E3 protein, a known PKR antagonist, during abortive replication (61). Similarly, many dysregulated cellular processes observed during abortive infection may reflect downstream consequences of inhibited protein synthesis rather than direct effects of SAMD9. Further studies employing targeted gene KO are needed to establish causal relationships.

Studies with mouse models have shown that SAMD9L is a tumor suppressor, with its haploinsufficiency linked to the development of myeloid malignancies (79). These studies also suggested that SAMD9L facilitates endosome fusion, as its absence was associated with defects in early endosome fusion and impaired growth receptor degradation. However, the role and significance of SAMD9/9L in regulating endosome fusion remain unresolved. Most evidence indicates that SAMD9/9L is confined to the cytosol, and studies of human cells with SAMD9 mutations have reported only subtle changes in endosome size, raising questions about their direct involvement in endosomal processes (80, 81).

### 3.4. Cellular Functions Associated with Human SAMD9/9L Mutations

Interest in SAMD9/9L surged after 2016, when germline mutations were linked to severe human disorders (82, 83). Germline mutations in SAMD9 were first linked to MIRAGE syndrome, a rare multisystem developmental disorder characterized by growth restriction, adrenal insufficiency, and other severe symptoms (84). Mutations in SAMD9L were initially associated with ataxia-pancytopenia syndrome, a condition marked by progressive neurological symptoms (85, 86). Subsequent studies expanded the disease spectrum, revealing that SAMD9/9L mutations contribute significantly to inherited bone marrow failure syndromes and pediatric myelodysplastic syndromes (87–90). Additionally, certain frameshift mutations in SAMD9L that truncate its C-terminal region have been linked to severe autoinflammatory diseases (91, 92).

**Figure 3:**
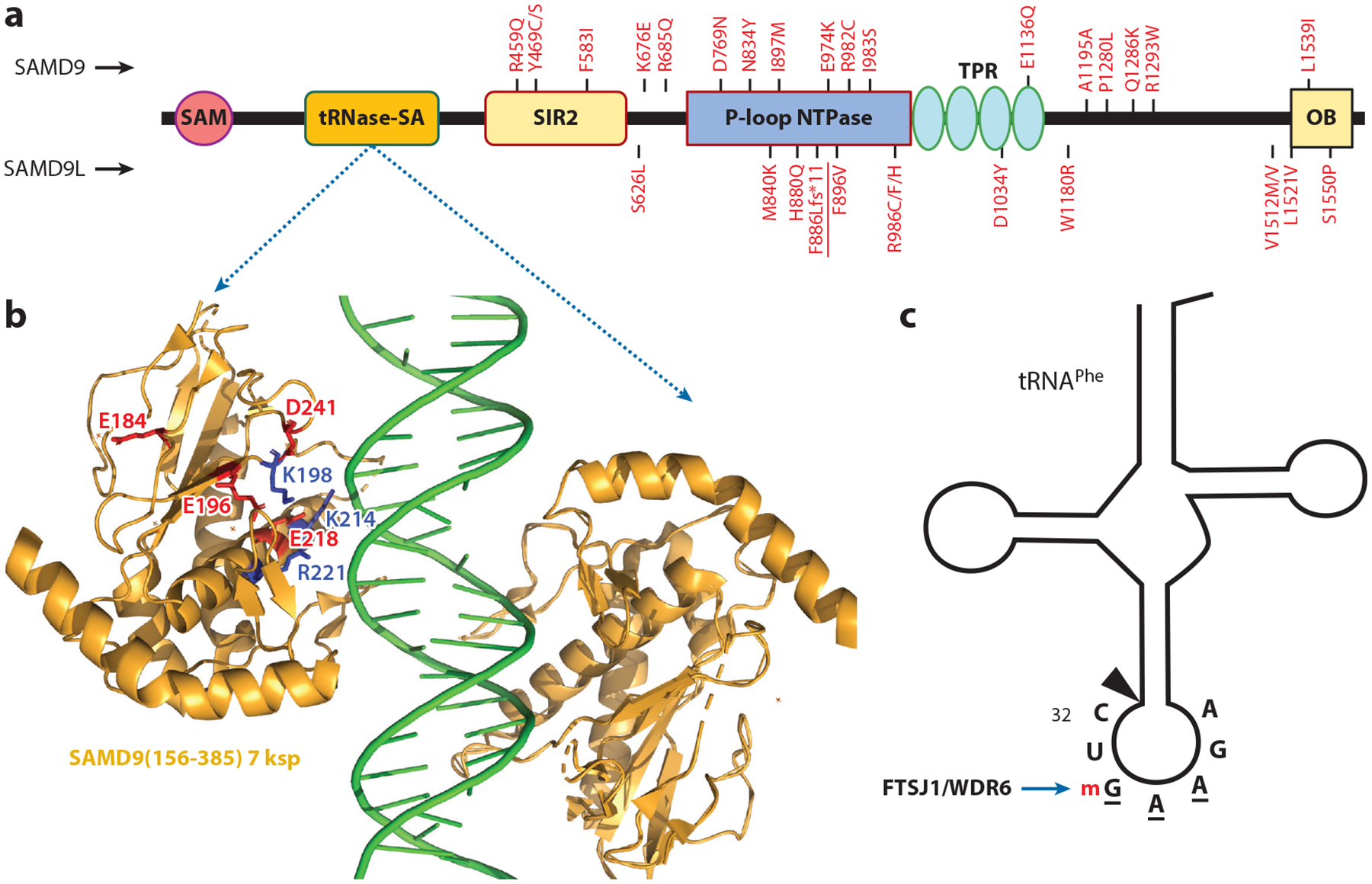
SAMD9/9L structure and function. (*a*) Predicted SAMD9/9L domain architecture with the following domains from N to C terminus: SAM, tRNase-SA, SIR2, P-loop NTPase, TPR, and OB domains. Representative patient-derived GoF mutations are indicated for SAMD9 (*above*) and SAMD9L (*below*). (*b*) The crystal structure of the hSAMD9 tRNase-SA domain (residues 156–385) in complex with double-stranded DNA, showing key residues responsible for nucleic acid interactions (K198, K214, R221) and catalytic activity (E184, E196, E218, D241). (*c*) tRNA^Phe^, the substrate of hSAMD9, is depicted with the sequence of the anticodon loop shown. The anticodon sequence (GAA) is underlined, and the 2′-O m at the wobble position is catalyzed by FTSJ1/WDR6. The cleavage site is marked by an arrow. Abbreviations: GoF, gain-of-function; hSAMD9, human SAMD9; m, methylation; OB, oligonucleotide/oligosaccharide binding; SAM, sterile alpha motif; SAMD9/9L, sterile alpha motif domain-containing 9/L; SIR2, silent information regulator 2; TPR, tetratricopeptide repeat; tRNA, transfer RNA; tRNase-SA, tRNA endoribonuclease SAMD9/9L.

Key discoveries from these studies were that overexpression of SAMD9 or SAMD9L reduces cell growth and that associated diseases are primarily driven by heterozygous gain-of-function (GoF) mutations (Figure 3), which enhance the growth inhibitory activity of the wild-type (WT) alleles (84, 87). A notable consequence of these GoF mutations is the frequent loss of the chromosome 7 copy harboring the mutated SAMD9/9L alleles in bone marrow cells. This process, known as monosomy 7, mitigates the growth-restrictive effects of the mutations but predisposes affected individuals to myeloid malignancies (82). Additionally, germline mutations that result in loss of function or haploinsufficiency of SAMD9/9L are associated with myeloid neoplasms (93–95), consistent with mouse model studies (79). To date, nearly 100 distinct disease-causing variants of SAMD9 and approximately 70 for SAMD9L have been reported (87), with the majority being GoF mutations.

GoF mutants have proved valuable for investigating the biological functions of SAMD9/9L without the confounding influence of viral infections. Because SAMD9/9L restrict poxvirus replication by inhibiting protein synthesis, the effects of GoF SAMD9/9L mutants on cellular protein synthesis had been specifically examined. Overexpression of WT SAMD9/9L was shown to inhibit cellular protein synthesis, an effect enhanced by GoF mutations (81, 92, 96, 97). The inhibition appears to target the elongation stage of protein synthesis (92, 96). These studies implicate translational control as a major mechanism underlying both the antiviral and antiproliferative activities of SAMD9/9L.

### 3.5. SAMD9/9L Structure and Function

Structural and functional studies recently revealed human SAMD9 as a poxvirus-activatable anticodon nuclease that selectively cleaves tRNA^Phe^, leading to phenylalanine-codon-specific ribosomal stalling, global inhibition of protein synthesis, and proteotoxic stress responses (96, 98).

#### 3.5.1. Predicted domain architecture.

SAMD9/9L are large cytoplasmic proteins with more than 1,500 aa. Initial sequence analysis only identified a sterile alpha motif (SAM) domain at the N terminus, which led to the naming of these proteins. Subsequently, more advanced sequence analysis techniques uncovered additional domains (74). The domains spanning from the N terminus to the C terminus are SAM, Alba (acetylation lowers binding affinity), SIR2 (silent information regulator 2), P-loop NTPase, TPR (tetratricopeptide repeat), and OB (oligonucleotide/oligosaccharide binding) (Figure 3a). Alba and OB domains are predicted to bind DNA and RNA, respectively. Interestingly, the P-loop NTPase domain contains a rare aa substitution in the P-loop (Walker A motif): A glycine replaces the conserved lysine, which is typically required for NTP hydrolysis (74). This substitution likely allows the domain to bind NTPs without functioning as an active NTPase.

The overall domain organization of SAMD9/9L resembles the STAND (signal transduction ATPases with numerous associated domains) superfamily of immune sensors, which includes APAF-1 (apoptotic protease activating factor 1) and NLRs (nucleotide-binding domain, leucine-rich repeat containing proteins) (99, 100). STAND proteins share a tripartite domain architecture comprising a C-terminal sensor that detects pathogens or cellular damage, a central nucleotide-binding oligomerization domain (NOD) that mediates nucleotide-driven oligomerization, and an N-terminal effector that is allosterically activated to initiate downstream effects.

#### 3.5.2. Identification of SAMD9/9L as poxvirus-activatable transfer RNA endoribonucleases.

The presence of a putative DNA-binding Alba domain prompted investigations into whether this domain might serve as the sensor for cytoplasmic DNA generated during poxvirus replication. A larger domain of hSAMD9 (aa 156–385) that encompasses the predicted Alba domain was identified as a double-stranded nucleic acid (dsNA)-binding domain (96). The crystal structure of the domain in complex with dsDNA was solved, providing detailed insights into its nucleic acid–binding mechanism. The domain binds dsNA in a sequence-independent manner via electrostatic interactions with the phosphate backbone, with K198, K214, and R221 identified as key residues essential for both dsNA binding and antiviral activities (Figure 3b). Interestingly, mutations in these residues also abrogate the antiproliferative activities of the constitutively active GoF SAMD9/9L mutants, indicating that dsNA binding acts as an effector function rather than a sensing mechanism (96).

Further studies revealed that this domain is a novel transfer RNA (tRNA) endoribonuclease, which has been named tRNase-SA (tRNA endoribonuclease SAMD9/9L) (98). tRNase-SA is a metal-dependent nuclease with a putative catalytic site comprising residues E184, E196, E218, and D241 in hSAMD9 (Figure 3b). It cleaves tRNAs at the anticodon loop with remarkable specificity, targeting a stem-loop structure containing a 2′-O-methylated guanosine (Figure 3c). This modification, introduced by the FTSJ1/WDR6 complex, marks tRNA^Phe^ as the primary substrate (98). Once activated, hSAMD9 depletes cellular tRNA^Phe^, leading to ribosomal stalling at phenylalanine codons and proteotoxic stress responses (98). The tRNase activity is essential for the antiviral, antiproliferative, and protein synthesis inhibitory functions of SAMD9/9L, as mutations in the dsNA binding or catalytic residues of tRNase-SA abolish these functions (96, 98).

Beyond poxviruses, SAMD9/9L are implicated in antiviral defense against flavivirus (101), papillomavirus (102), and human immunodeficiency virus type 1 (HIV-1) (103, 104). A recent report indicates that hSAMD9L, but not SAMD9, functions as a restriction factor against HIV-1 (104), suggesting some functional divergence between these paralogs. The underlying mechanism involves suppression of protein synthesis, and mutations within the putative tRNase-SA catalytic site abolish the antiviral activities (104).

#### 3.5.3. Activation of tRNase-SA by poxvirus infection or gain-of-function mutations.

tRNase-SA in full-length SAMD9/9L is latent but becomes activated during poxvirus infection as early as two hours post-infection (98). The exact triggers for SAMD9/9L activation are currently unknown, but it is established that their activation does not require viral genome replication.

In the absence of infection, various patient-derived GoF mutations render tRNase-SA constitutively active (98), leading to protein synthesis shutdown and proteotoxic stress. These GoF mutations cluster in the central NTPase domain and the C-terminal half (Figure 3a), likely disrupting an autoinhibitory mechanism mediated by the C-terminal half. Overexpression of tRNA^Phe^ can partially rescue protein synthesis and reduce SAMD9/9Ľs antiviral and antiproliferative activities (98, 105).

**Figure 4:**
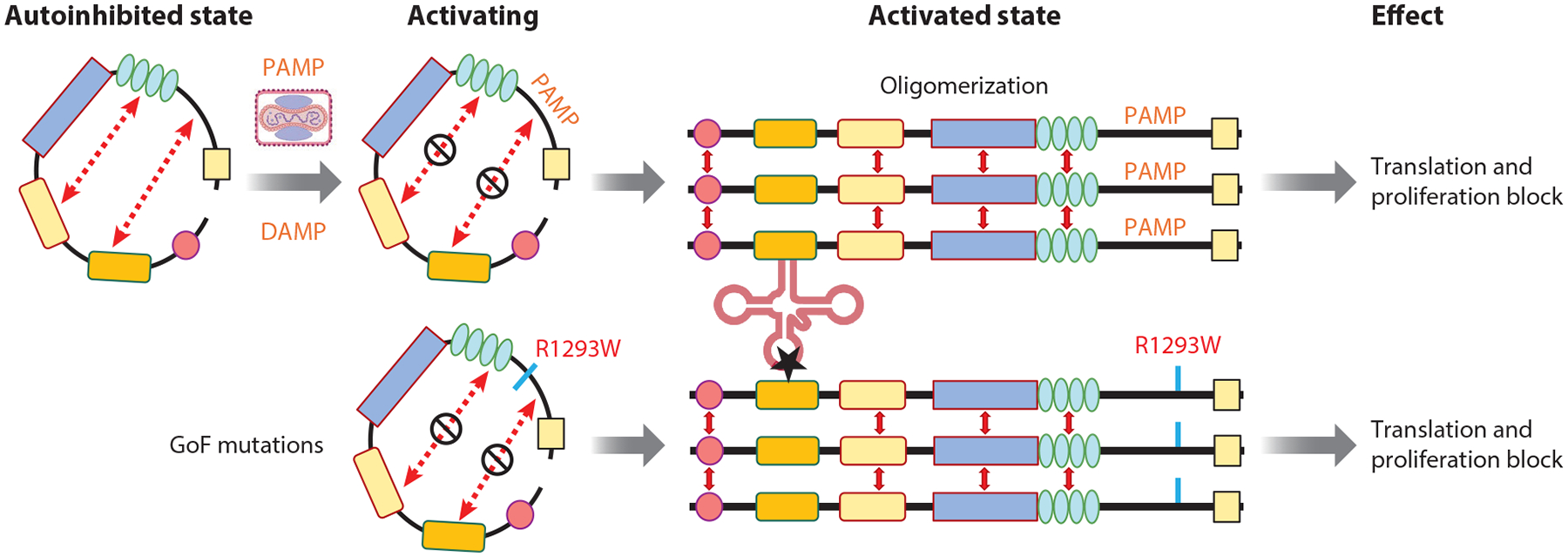
Model of SAMD9/9L regulation and dysregulation. Based on the mechanisms of other STAND family proteins, SAMD9/9L are believed to normally exist in a closed, inactive conformation stabilized by intramolecular interactions (*dashed lines*). Upon detection of PAMPs or DAMPs via their C-terminal sensors, they may undergo conformational changes, transitioning to an open, active state. This activation triggers their tRNase activity, leading to the inhibition of global protein synthesis as part of the antiviral or stress response. GoF mutations, such as R1297W, destabilize these intramolecular interactions, shifting the equilibrium toward a constitutively active state, resulting in unregulated tRNase activity and subsequent cellular dysfunction. Abbreviations: DAMP, damage-associated molecular pattern; GoF, gain-of-function; PAMP, pathogen-associated molecular pattern; SAMD9/9L, sterile alpha motif domain-containing 9/L.

Based on these studies, a model of SAMD9/9L activation analogous to STAND protein signaling (99) can be envisioned (Figure 4). In this model, SAMD9/9L predominantly exist in an off state, maintained by intramolecular interactions. Upon sensing PAMPs or damage-associated molecular patterns (DAMPs), SAMD9/9L may undergo conformational changes, adopting open, active oligomeric forms that initiate tRNase-SA activity and inhibit protein synthesis. GoF mutations such as R1297W may disrupt intramolecular interactions, relieving autoinhibition and driving constitutive tRNase-SA activity.

#### 3.5.4. Evolutionary connections to prokaryotic antiviral STAND proteins.

tRNase-SA is classified as a tRNA anticodon nuclease that is employed by microbes to defend against viruses or competitors but has not been previously observed in multicellular eukaryotes (106). The utilization of tRNase-SA as the effector function by SAMD9/9L is a departure from previously characterized eukaryotic STAND proteins, which all recruit adaptors and require secondary signaling to exert their effect (100). This intrinsic effector mechanism draws a surprising parallel to prokaryotic antiviral STAND (AVS) proteins that have been recently discovered (107). AVS proteins use diverse N-terminal nucleases as direct effectors and C-terminal sensors to recognize a variety of phage proteins, including conserved structural elements such as phage terminase (107, 108). Emerging evidence suggests that components of eukaryotic innate immunity may have been acquired via horizontal gene transfer from prokaryotic antiphage systems, underscoring the conservation of immune defense strategies across domains of life (109).

## 4. POXVIRUS INHIBITORS OF SAMD9/9L

SAMD9 and SAMD9L are targeted by three distinct poxvirus host-range factors: K1, C7, and CP77. All OPXVs encode at least one of these inhibitors, while many mammalian poxviruses outside the OPXV genus encode C7 homologs (17, 61). Species-specific divergence in SAMD9/9L results in differential susceptibility to these viral antagonists (14, 15).

### 4.1. Poxvirus C7 Family

Nearly all mammalian poxviruses encode at least one host-range factor homologous to VACV C7. Members of the C7 family fall into two functional classes: bona fide antagonists that bind and inhibit SAMD9/9L and homologs that lack measurable SAMD9/9L-inhibitory activity.

#### 4.1.1. Distribution of C7 family in poxviruses.

VACV C7 is a 150-aa protein with no known homologs outside poxviruses. In OPXV, C7 has a paralog, known as CPXV020 in CPXV. Both C7 and CPXV020 are encoded in the variable terminal regions of the genome. C7 is conserved across OPXV species, whereas CPXV020 is retained in some OPXV species but is fragmented or lost in others, including VACV (Figure 2b). Outside OPXVs, C7 homologs are present in several mammalian poxvirus genera (61, 69), located within the central genomic region. Notably, MYXV encodes three tandemly located C7 homologs: M62, M63, and M64.

C7 homologs were initially characterized by their ability to rescue VACV/K1^−^C7^−^ replication and classified into two groups (61, 69). Functional homologs, including MYXV M62, sheeppox virus (SPPV) 063, and swinepox virus 064, can support replication, while nonfunctional homologs, including CPXV020, MYXV M63, and MYXV M64, cannot.

Previous phylogenetic analysis of C7 homologs revealed three clades represented by VACV C7, CPXV020, and MYXV M62 (110). Members of these clades are defined by sequence homology and gene synteny. Additional C7 homologs were also noted, though their functions have not been characterized. COTV, for instance, encodes two C7 homologs: one resembling MYXV M62, found in the central genomic region, and another resembling VACV C7, located in the terminal region of the genome, possibly due to a recombination event with OPXV.

Interestingly, a newly emergent MYXV strain (MYXV-Tol), responsible for lethal outbreaks in Iberian hares, has acquired an additional M62-like homolog (M159) through recombination with an unidentified poxvirus (6). Deleting M159 abolishes MYXV-Tol replication in hare cells, demonstrating how acquiring a single C7-like factor can facilitate host jumps.

C7 homologs were once thought to be relevant only for mammalian poxviruses (61). However, recent genome sequencing of avipoxviruses has revealed a distinct fourth clade of C7 homologs, defined by shared synteny and sequence homology (Figure 2b,c). This C7 homolog is known as gp159 in turkeypox virus, a basal avipoxvirus lineage (111). It is also present in a few other avipoxviruses such as pigeonpox virus but is missing in fowlpox virus and canarypox virus. This suggests that avian SAMD9/9L homologs may also restrict poxviruses and that avipoxviruses may have evolved specific inhibitors against avian SAMD9/9L homologs.

#### 4.1.2. Structure and function of the C7 family.

Among the four recognized C7 clades, VACV C7 and certain MYXV M62 clade members have been shown to support VACV in human cells and bind hSAMD9/9L (16, 17, 112). In contrast, CPXV020 and some MYXV M62 clade members, such as M63 and M64, cannot perform these functions (61, 112). Studies of MYXV indicate that M64 is not a host-range factor for MYXV (113), while M62 and M63 play a different role for MYXV host range. M62 is essential for MYXV replication in all cell types (17), while M63 is necessary only for replication and pathogenesis in rabbits (114). M62 and M63 form a complex that enhances SAMD9 binding (17).

**Figure 5:**
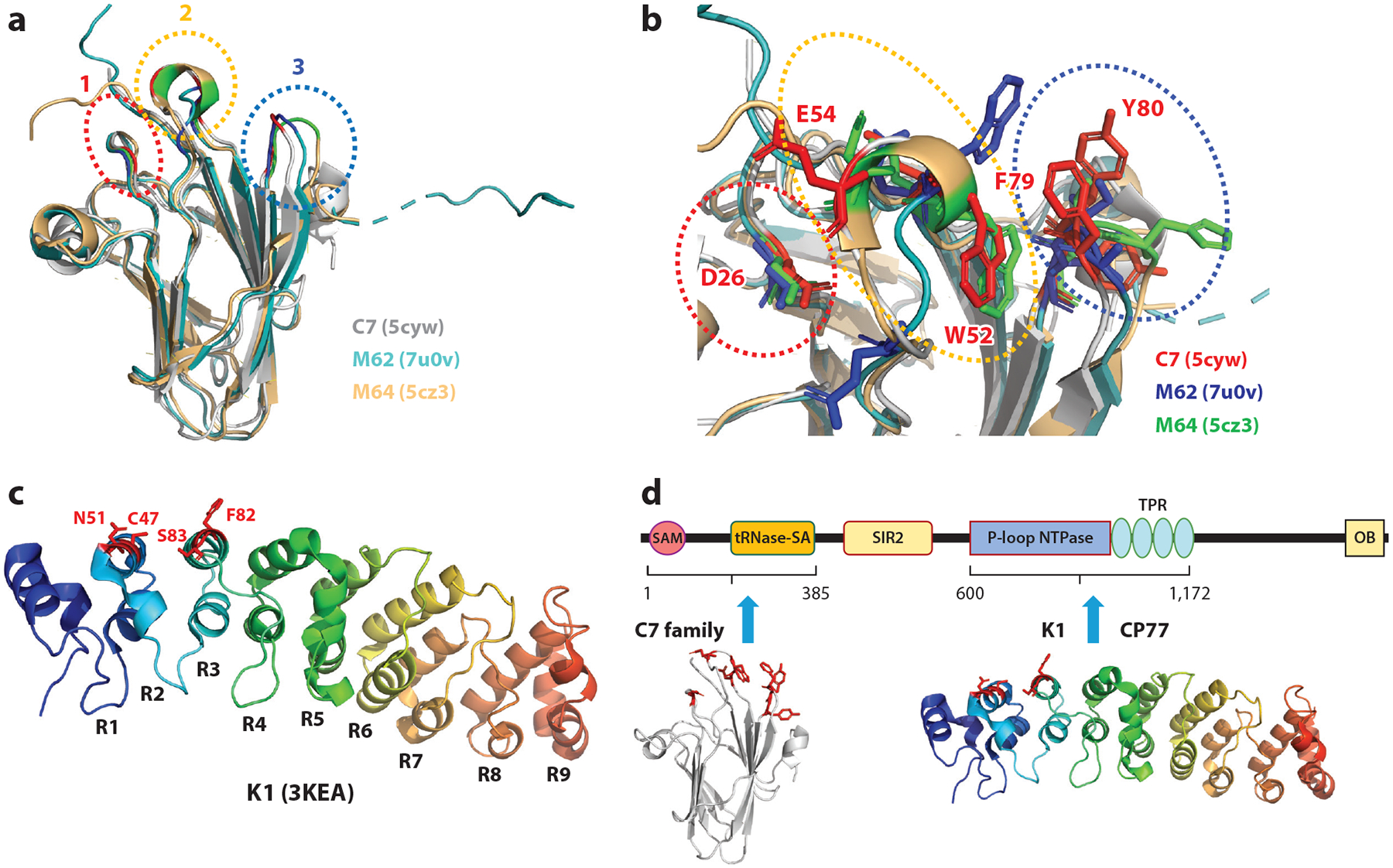
Molecular basis of SAMD9/9L inhibition by poxvirus host-range factors. (*a*) Structural alignment of three C7 family members: VACV C7 (PDB: 5cyw), MYXV M62 (PDB: 7u0v), and MYXV M64 (PDB: 5cz3). All share a conserved b-sandwich fold, with variations in the C-terminal tails. The three-fingered molecular claw, composed of three surface loops responsible for SAMD9/9L binding, is labeled. (*b*) The C7 family members are distinguished by sequence variations in the molecular claws. This zoomed-in view from panel *a* shows key residues in the molecular claws of the three proteins. Key residues in VACV C7 are labeled. (*c*) VACV K1 adopts a curved structure composed of nine ANK repeats. Residues essential for its host-range function (C47, N51, F82, and S83) are marked on the structure. (*d*) C7 family proteins primarily target the N-terminal 385 amino acids of SAMD9, whereas K1 and CP77 interact with the central region of SAMD9 (~607–1172), which includes the P-loop NTPase and TPR domains. Abbreviations: ANK, ankyrin; MYXV, myxoma virus; OB, oligonucleotide/oligosaccharide binding; PDB, Protein Data Bank; SAM, sterile alpha motif; SAMD9/9L, sterile alpha motif domain-containing 9/L; SIR2, silent information regulator 2; TPR, tetratricopeptide repeat; tRNase-SA, tRNA endoribonuclease SAMD9/9L; VACV, vaccinia virus.

The crystal structures of VACV C7 and some MYXV homologs have been determined, revealing a shared b-sandwich fold with variable C-terminal regions (112) (Figure 5a). Three loops on the surface of the b-sandwich form a “three-fingered molecular claw” for binding SAMD9/9L (112, p. 14859). These loops have unique electrostatic and hydrophobic properties (112). The M62 and M64 structures exhibit variations in this molecular claw, which could influence their SAMD9/9L binding. M62 can bind directly to the N-terminal 385 aa of SAMD9 (115), indicating that C7 homologs target the SAM and tRNase SA domains.

Most functional C7 homologs can inhibit both hSAMD9 and murine SAMD9L (mSAMD9L). However, the SPPV C7 homolog demonstrates reduced binding to mSAMD9L and cannot support VACV replication in mouse cells (15, 69). This specificity is attributed to a 2-aa difference (N134, F135) near the molecular claw, which impairs mSAMD9L interaction while retaining activity against hSAMD9/9L (15). These findings underscore the role of subtle structural perturbation of the molecular claw in mediating species-specific SAMD9/9L inhibition.

### 4.2. K1 and CP77

In addition to C7, OPXVs encode two other SAMD9/9L inhibitors, K1 and CP77. Both K1 and CP77 are structurally unrelated to C7 and bind SAMD9/9L at distinct, nonoverlapping regions.

#### 4.2.1. Distribution of K1 and CP77 in poxviruses.

*K1L* and *CP77* are OPXV-specific genes that have been selectively retained in different OPXV species (Figure 2b). For example, MPXV and CPXV retain both genes, whereas VACV has lost *CP77* and VARV has lost both *K1L* and *CP77*. The retention of three SAMD9/9L inhibitors (K1, C7, and CP77) in species endemic in wild rodents (CPXV, MPXV, and North American OPXVs) suggests a need for multiple inhibitors to counteract diverse SAMD9/9L in rodent hosts (14).

Interestingly, genome sequencing of ancient VARV strains (aVARV), widespread in northern Europe in the Viking Age, reveals that *K1L* was present in these earlier lineages but was only lost after the divergence of the aVARV and modern VARV clades approximately 1,700 years ago (116). This and other gene loss coincide with VARV's increased virulence in humans (117).

Outside OPXV, CETV and COTV also encode K1, likely acquired through recombination with OPXV. Notably, NY_014 poxvirus has two K1 homologs (118). This illustrates the complex evolutionary relationships among poxviruses and their capacity for horizontal gene transfer.

#### 4.2.2. Structure and function of K1 and CP77.

K1 and CP77 function equivalently to C7 in supporting VACV replication in many cell lines, although differences have been observed in certain host cell types. For instance, K1 or CP77, but not C7, can support VACV replication in rabbit RK13 cells, while only CP77 can support replication in Chinese hamster ovary (CHO) cells (59). This difference is likely due to host-specific differences in SAMD9/9L. In CHO cells, Chinese hamster SAMD9L (chSAMD9L) acts as the dominant restriction factor and is resistant to inhibition by K1 and C7 but is susceptible to CP77 (14), explaining the long-standing puzzle of why CHO cells are nonpermissive for VACV but permissive for CPXV (14). This also demonstrates that host species-specific differences in SAMD9L pose a barrier to cross-species poxvirus infection.

VACV K1 is a 284-aa protein. The K1 structure has been solved, revealing that it is composed entirely of 9 ANK repeats (119). Residues critical for K1 function in human cells have been mapped to Asn51, Cys47, Phe82, and Ser83, which are on two consecutive ANK repeats (Figure 5c), forming a continuous surface. These residues are also critical for K1 binding with hSAMD9 (14), demonstrating that K1's host-range function in human cells correlates with its binding to SAMD9. Intriguingly, K1's function in rabbit RK13 cells requires a larger number of ANK repeats than in human cells (63, 119), reflecting a host species-specific requirement for K1.

CPXV CP77 is a 668-aa protein. CP77 is predicted to contain 9 ANK repeats and a C-terminal PRANC domain. Only the N-terminal 352 residues of CP77 are required for VACV growth in CHO cells, and the deletion of ANK repeat 5 could disrupt CP77 function (65). Correspondingly, only the N-terminal 382 aa is essential for chSAMD9L binding, while deletion of ANK repeat 5 abolishes the binding, demonstrating that the host-range function of CP77 in CHO cells correlates with its binding to chSAMD9L (14). Furthermore, the ability of OPXV species to antagonize chSAMD9L and grow in CHO cells correlates with their encoding a CP77 ortholog. OPXV species that encode a full-length CP77 ortholog, including MPXV, can replicate in both the parental and SAMD9L KO CHO cells, while OPXV species that have lost the CP77 gene can replicate only in SAMD9L KO CHO cells (14).

hSAMD9/9L aa ~607–1172 are sufficient for binding to both K1 and CP77 (14), indicating that K1 and CP77 target the same region of SAMD9/9L, which contains the putative NTPase and TPR domains. Notably, this region is distinct from the one targeted by the C7 family.

## 5. CONCLUSION AND FUTURE DIRECTIONS

As conspicuous cytoplasmic pathogens, poxviruses must overcome nearly all facets of the host innate immune system to replicate successfully. This extraordinary challenge has made poxviruses invaluable models for studying host-pathogen interactions and innate immunity. Over the past decade, studies of poxvirus host-range mutants have revealed novel antiviral factors, such as SAMD9/9L, and unexpected mechanisms within established immune pathways. Continued research in this area will further illuminate the complexities of host-pathogen interactions and innate immunity.

With respect to SAMD9/9L and their viral inhibitors, many important questions remain unanswered. The critical role of SAMD9/9L in restricting poxvirus replication is evident from the complete cessation of viral replication when these factors are not antagonized and the existence of multiple, mutually redundant poxvirus inhibitors targeting distinct regions of SAMD9/9L. However, it is puzzling that SAMD9/9L do not exhibit similarly potent antiviral effects against other viruses. This discrepancy suggests that poxviruses may produce unique PAMPs that activate SAMD9/9L or that other viruses may encode as-yet-undiscovered SAMD9/9L inhibitors—or possibly both. Elucidating the mechanisms by which SAMD9/9L are activated is thus a critical research priority. Such findings could identify additional viruses susceptible to SAMD9/9L restriction and reveal how DAMPs in dysregulated tumor cells trigger SAMD9/9Ľs tumor suppressor functions.

The broad distribution of SAMD9/9L across animal species also presents opportunities to explore their evolutionary conservation and functional divergence. For instance, avian poxviruses are restricted to birds, while mammalian poxviruses infect only mammals. While host specificity likely arises from a combination of factors, species-specific differences in SAMD9/9L may contribute significantly to these patterns. Moreover, understanding how poxviruses lacking known SAMD9/9L inhibitors evade these restriction factors may reveal novel evasion strategies and deepen our understanding of virus-host coevolution. The interplay between SAMD9/9L and their viral antagonists exemplifies the dynamic arms race between host defenses and pathogen adaptations. Studying these interactions not only enriches our understanding of species-specific infection barriers but also advances our knowledge of how innate immunity evolves and responds to emerging viral threats.

## Figures and Tables

**Table 1:** Vertebrate *Poxviridae* genera and their representative species

Genus	Species
*Orthopoxvirus* (OPXV)	Cowpox virus: CPXV
	Taterapox virus: TATV
	Camelpox virus: CMLV
	mpox virus: MPXV
	Variola virus: VARV
	Vaccinia virus: VACV
	Ectromelia virus: ECTV
	Volepox virus: VPXV
	Skunkpoxvirus: SKPV
	Raccoonpox virus: RCNV
*Centapoxvirus*	NY_014 poxvirus: NY14
	Murmansk poxvirus: MMPV
	Yokapox virus: YKPV
*Suipoxvirus*	Swinepox virus: SWPV
*Capripoxvirus*	Sheeppox virus: SPPV
*Leporipoxvirus*	Myxoma virus: MYXV
*Cervidpoxvirus*	Deerpox virus: DPV
*Oryzopoxvirus*	Cotia virus: COTV
*Yatapoxvirus*	Yaba-like disease virus: YLDV
*Vespertiliopoxvirus*	Eptesipoxvirus: EPTV
*Pteropoxvirus*	Pteropox virus: PTPV
*Mustelpoxvirus*	Sea otterpox virus: SOPV
*Parapoxvirus*	Orf virus: ORFV
*Sciuripoxvirus*	Squirrelpox virus: SQPV
*Molluscipoxvirus*	Molluscum contagiosum virus: MOCV
*Macropopoxvirus*	Eastern gray kangaroo poxvirus: EKPV
*Avipoxvirus*	Penguinpox virus: PEPV
	Pigeonpox virus: PNPV
	Fowlpox virus: FWPV
	Canarypox virus: CNPV
	Turkeypox virus: TKPV
*Crocodylidpoxvirus*	Crocodylidpoxvirus: CRV
*Salmonpoxvirus*	Salmon-gill poxvirus: SMGPV
